# Diaqua­bis­(4-bromo­benzoato-κ*O*)bis­(*N*,*N*-diethyl­nicotinamide-κ*N* ^1^)copper(II)

**DOI:** 10.1107/S1600536811034787

**Published:** 2011-08-31

**Authors:** Hacali Necefoğlu, Füreya Elif Özbek, Vijdan Öztürk, Vedat Adıgüzel, Tuncer Hökelek

**Affiliations:** a Department of Chemistry, Kafkas University, 36100 Kars, Turkey; b Department of Physics, Hacettepe University, 06800 Beytepe, Ankara, Turkey

## Abstract

The title Cu^II^ complex, [Cu(C_7_H_4_BrO_2_)_2_(C_10_H_14_N_2_O)_2_(H_2_O)_2_], contains two 4-bromo­benzoate (PBB), two diethyl­nicotinamide (DENA) monodentate ligands and two water mol­ecules. The four O atoms in the equatorial plane around the Cu^II^ ion form a slightly distorted square-planar arrangement, while the slightly distorted octa­hedral coordination is completed by two N atoms of the DENA ligands in the axial positions. Intra­molecular O—H⋯O hydrogen bonds link the water mol­ecules to the carboxyl­ate groups. The dihedral angles between the carboxyl­ate groups and the adjacent benzene rings are 3.1 (3) and 3.74 (17)°, while the pyridine rings and the benzene rings are oriented at dihedral angles of 6.81 (10) and 3.38 (12)°. In the crystal, inter­molecular O—H⋯O hydrogen bonds link the mol­ecules into double chains along the *b* axis. C—H⋯O inter­actions are also observed. π–π contacts between pyridine rings [centroid–centroid distance = 3.485 (2) Å] may further stabilize the crystal structure.

## Related literature

For literature on niacin, see: Krishnamachari (1974 ▶). For information on the nicotinic acid derivative *N*,*N*-diethyl­nicotinamide, see: Bigoli *et al.* (1972 ▶). For related structures, see: Hökelek *et al.* (1996 ▶, 2009*a* ▶,*b* ▶); Hökelek & Necefoğlu (1998 ▶, 2007 ▶); Necefoğlu *et al.* (2011 ▶). For bond-length data, see: Allen *et al.* (1987 ▶).
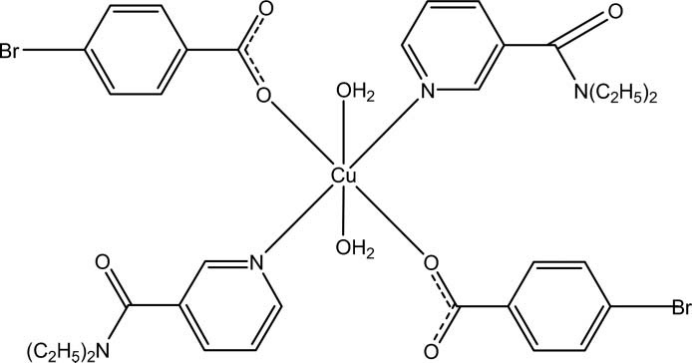


## Experimental

### 

#### Crystal data

[Cu(C_7_H_4_BrO_2_)_2_(C_10_H_14_N_2_O)_2_(H_2_O)_2_]
*M* *_r_* = 856.05
Monoclinic, 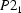

*a* = 8.3621 (2) Å
*b* = 12.2183 (3) Å
*c* = 17.6504 (4) Å
β = 101.478 (3)°
*V* = 1767.29 (8) Å^3^
*Z* = 2
Mo *K*α radiation
μ = 2.94 mm^−1^
*T* = 100 K
0.41 × 0.18 × 0.12 mm

#### Data collection

Bruker Kappa APEXII CCD diffractometer
Absorption correction: multi-scan (*SADABS*; Bruker, 2005 ▶) *T*_min_ = 0.536, *T*_max_ = 0.703
17073 measured reflections
7011 independent reflections
6116 reflections with *I* > 2σ(*I*)
*R*_int_ = 0.036

#### Refinement

*R*[*F* ^2^ > 2σ(*F* ^2^)] = 0.035
*wR*(*F* ^2^) = 0.079
*S* = 1.03
7011 reflections
463 parameters
5 restraints
H atoms treated by a mixture of independent and constrained refinement
Δρ_max_ = 1.08 e Å^−3^
Δρ_min_ = −0.59 e Å^−3^
Absolute structure: Flack (1983 ▶), 2353 Friedel pairs
Flack parameter: 0.412 (7)

### 

Data collection: *APEX2* (Bruker, 2007 ▶); cell refinement: *SAINT* (Bruker, 2007 ▶); data reduction: *SAINT*; program(s) used to solve structure: *SHELXS97* (Sheldrick, 2008 ▶); program(s) used to refine structure: *SHELXL97* (Sheldrick, 2008 ▶); molecular graphics: *ORTEP-3 for Windows* (Farrugia, 1997 ▶); software used to prepare material for publication: *WinGX* (Farrugia, 1999 ▶) and *PLATON* (Spek, 2009 ▶).

## Supplementary Material

Crystal structure: contains datablock(s) I, global. DOI: 10.1107/S1600536811034787/su2303sup1.cif

Structure factors: contains datablock(s) I. DOI: 10.1107/S1600536811034787/su2303Isup2.hkl

Additional supplementary materials:  crystallographic information; 3D view; checkCIF report

## Figures and Tables

**Table 1:** Hydrogen-bond geometry (Å, °)

*D*—H⋯*A*	*D*—H	H⋯*A*	*D*⋯*A*	*D*—H⋯*A*
O7—H71⋯O2	0.83 (2)	1.93 (3)	2.692 (4)	154 (5)
O7—H72⋯O4^i^	0.83 (4)	2.04 (4)	2.834 (4)	163 (3)
O8—H81⋯O4	0.84 (2)	1.88 (2)	2.702 (4)	170 (4)
O8—H82⋯O6^ii^	0.82 (4)	2.05 (4)	2.848 (4)	168 (5)
C11—H11⋯O5^iii^	0.93	2.41	3.106 (5)	133
C16—H16⋯O4^iii^	0.93	2.46	3.359 (5)	162
C26—H26⋯O6^iv^	0.93	2.37	3.261 (4)	160

Symmetry codes: (i) 
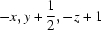
; (ii) 
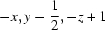
; (iii) 
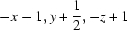
; (iv) 
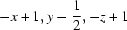
.

**Fig. 1.**
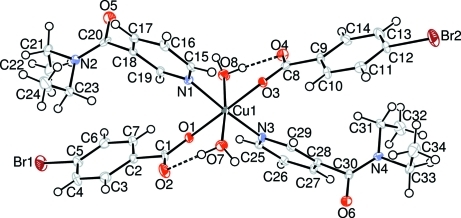
The molecular structure of the title molecule with the atom-numbering scheme. Displacement ellipsoids are drawn at the 50% probability level. The intramolecular O—H···O hydrogen bonds are shown as dashed lines [see Table 1 for details].

**Fig. 2.**
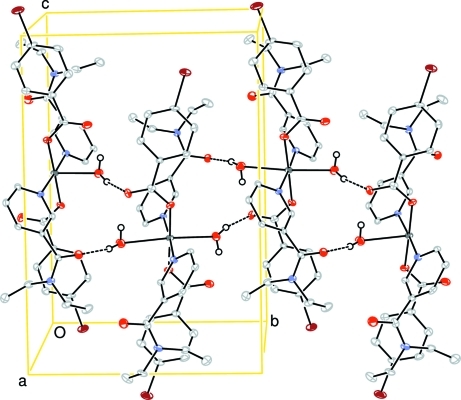
A view along the a-axis of the crystal packing of the title compound. Only the intermolecular O—H···O hydrogen bonds are shown as dashed lines [see Table 1 for details; H-atoms not involved in hydrogen bonding have been omitted for clarity].

## supplementary crystallographic information

### Comment

As a part of our ongoing investigations of transition metal complexes of nicotinamide (NA), one form of niacin (Krishnamachari, 1974), and/or the nicotinic acid derivative *N*,*N*-diethylnicotinamide (DENA), an important respiratory stimulant (Bigoli *et al.*, 1972), the title compound was synthesized and its crystal structure is reported on herein.

The title complex, (Fig. 1), is a mononuclear Cu^II^complex, consisting of two *N*,*N*-diethylnicotinamide (DENA), two 4-bromobenzoate (PBB) ligands and two coordinated water molecules, all ligands coordinating in a monodentate manner. The crystal structures of similar omplexes of Cu^II^, Co^II^, Ni^II^, Mn^II^ and Zn^II^ ions, [Cu(C_7_H_5_O_2_)_2_(C_10_H_14_N_2_O)_2_] (Hökelek *et al.*, 1996), [Co(C_6_H_6_N_2_O)_2_(C_7_H_4_NO_4_)_2_(H_2_O)_2_] (Hökelek & Necefoğlu, 1998), [Co(C_9_H_9_O_2_)_2_(C_10_H_14_N_2_O)_2_(H_2_O)_2_] (Necefoğlu *et al.*, 2011), [Ni(C_7_H_4_ClO_2_)_2_(C_6_H_6_N_2_O)_2_(H_2_O)_2_] (Hökelek *et al.*, 2009*a*), [Mn(C_9_H_10_NO_2_)_2_(H_2_O)_4_].2H_2_O (Hökelek & Necefoğlu, 2007) and [Zn(C_7_H_4_BrO_2_)_2_(C_6_H_6_N_2_O)_2_(H_2_O)_2_] (Hökelek *et al.*, 2009*b*) have also been reported. In the copper(II) complex mentioned above the two benzoate ions coordinate to the Cu^II^ atom as bidentate ligands, while in the other structures all the ligands coordinate in a monodentate manner.

In the title complex (Fig. 1), the four O atoms (O1, O3, O7 and O8) in the equatorial plane around the Cu^II^ ion form a slightly distorted square-planar arrangement, while the slightly distorted octahedral coordination is completed by the two N atoms of the DENA ligands (N1 and N3) in the axial positions. The intramolecular O—H···O hydrogen bonds link the water molecules to the carboxylate groups (Table 1). The near equalities of the C1—O1 [1.289 (4) Å], C1—O2 [1.200 (5) Å] and C8—O3 [1.277 (4) Å], C8—O4 [1.229 (4) Å] bonds in the carboxylate groups indicate delocalized bonding arrangements, rather than localized single and double bonds. The Cu—O bond lengths are 1.985 (2) and 1.979 (2) Å (for benzoate oxygens) and 2.377 (3) and 2.543 (3) Å (for water oxygens), and the Cu—N bond lengths are 1.996 (3) and 1.998 (3) Å, close to standard values (Allen *et al.*, 1987). The Cu atom is displaced out of the least-squares planes of the carboxylate groups (O1/C1/O2) and (O3/C8/O4) by -0.7206 (4) and 0.6797 (4) Å, respectively. The dihedral angles between the planar carboxylate groups and the adjacent benzene rings A (C2—C7) and B (C9—C14) are 3.06 (28) and 3.74 (17) °, respectively. The benzene A (C2—C7) and B (C9—C14) rings and the pyridine C (N1/C15—C19) and D (N3/C25—C29) rings are oriented at dihedral angles of A/B = 3.38 (12), A/C = 67.74 (11), A/D = 61.26 (11), B/C = 67.57 (11), B/D = 61.24 (11) and C/D = 6.81 (10) °.

In the crystal, intermolecular O—H···O hydrogen bonds link the molecules into double chains along the b-axis (Table 1 and Fig. 2). There also exist C—H···O interactions. The π–π contact between the pyridine rings, Cg3—Cg4^i^, [symmetry code: (i) 1 + x, y, z, where Cg3 and Cg4 are centroids of the rings C (N1/C15—C19) and D (N3/C25—C29), respectively], may further stabilize the structure, with centroid-centroid distance of 3.485 (2) Å.

### Experimental

The title compound was prepared by the reaction of CuSO_4_.5H_2_O (1.23 g, 5 mmol) in H_2_O (20 ml) and DENA (1.78 g, 10 mmol) in H_2_O (20 ml) with sodium 4-bromobenzoate (2.23 g, 10 mmol) in H_2_O (50 ml) at room temperature. The mixture was filtered and set aside to crystallize at ambient temperature for two weeks, giving blue single crystals.

### Refinement

The compound crystallized as an inversion twin: refined BASF parameter = 0.412 (7), for 2353 Friedel pairs (50.5% coverage). Atoms H71, H72, H81 and H82 (for water molecules) were located in a difference Fourier map and were freely refined. The C-bound H-atoms were positioned geometrically with C—H = 0.93, 0.97 and 0.96 Å, for aromatic, methylene and methyl H-atoms, respectively, and constrained to ride on their parent atoms, with *U*_iso_(H) = k × *U*_eq_(C), where k = 1.5 for methyl H-atoms and k = 1.2 for all other H-atoms.

### Crystal data

**Table:**

[Cu(C_7_H_4_BrO_2_)_2_(C_10_H_14_N_2_O)_2_(H_2_O)_2_]	*F*(000) = 870
*M**_r_* = 856.05	*D*_x_ = 1.609 Mg m^−^^3^
Monoclinic, *P*2_1_	Mo *K*α radiation, λ = 0.71073 Å
Hall symbol: P 2yb	Cell parameters from 6540 reflections
*a* = 8.3621 (2) Å	θ = 2.4–27.7°
*b* = 12.2183 (3) Å	µ = 2.94 mm^−^^1^
*c* = 17.6504 (4) Å	*T* = 100 K
β = 101.478 (3)°	Block, blue
*V* = 1767.29 (8) Å^3^	0.41 × 0.18 × 0.12 mm
*Z* = 2	

### Data collection

**Table:**

Bruker Kappa APEXII CCD area-detector diffractometer	7011 independent reflections
Radiation source: fine-focus sealed tube	6116 reflections with *I* > 2σ(*I*)
graphite	*R*_int_ = 0.036
φ and ω scans	θ_max_ = 28.4°, θ_min_ = 2.0°
Absorption correction: multi-scan (*SADABS*; Bruker, 2005)	*h* = −11→11
*T*_min_ = 0.536, *T*_max_ = 0.703	*k* = −13→16
17073 measured reflections	*l* = −23→19

### Refinement

**Table:**

Refinement on *F*^2^	Secondary atom site location: difference Fourier map
Least-squares matrix: full	Hydrogen site location: inferred from neighbouring sites
*R*[*F*^2^ > 2σ(*F*^2^)] = 0.035	H atoms treated by a mixture of independent and constrained refinement
*wR*(*F*^2^) = 0.079	*w* = 1/[σ^2^(*F*_o_^2^) + (0.040*P*)^2^ + 0.1414*P*] where *P* = (*F*_o_^2^ + 2*F*_c_^2^)/3
*S* = 1.03	(Δ/σ)_max_ = 0.002
7011 reflections	Δρ_max_ = 1.08 e Å^−^^3^
463 parameters	Δρ_min_ = −0.59 e Å^−^^3^
5 restraints	Absolute structure: Flack (1983), 2353 Friedel pairs
Primary atom site location: structure-invariant direct methods	Flack parameter: 0.412 (7)

### Special details

**Table:**

Geometry. All esds (except the esd in the dihedral angle between two l.s. planes) are estimated using the full covariance matrix. The cell esds are taken into account individually in the estimation of esds in distances, angles and torsion angles; correlations between esds in cell parameters are only used when they are defined by crystal symmetry. An approximate (isotropic) treatment of cell esds is used for estimating esds involving l.s. planes.
Refinement. Refinement of F^2^ against ALL reflections. The weighted R-factor wR and goodness of fit S are based on F^2^, conventional R-factors R are based on F, with F set to zero for negative F^2^. The threshold expression of F^2^ > 2sigma(F^2^) is used only for calculating R-factors(gt) etc. and is not relevant to the choice of reflections for refinement. R-factors based on F^2^ are statistically about twice as large as those based on F, and R- factors based on ALL data will be even larger.

### Fractional atomic coordinates and isotropic or equivalent isotropic displacement parameters (Å^2^)

**Table:**

	*x*	*y*	*z*	*U*_iso_*/*U*_eq_	
Br1	0.09645 (6)	0.00081 (4)	1.08082 (2)	0.03240 (12)	
Br2	−0.29887 (6)	0.21016 (3)	0.08947 (2)	0.03089 (12)	
Cu1	−0.08109 (5)	0.12231 (3)	0.58764 (2)	0.01174 (10)	
O1	0.0327 (3)	0.1074 (2)	0.69709 (12)	0.0142 (5)	
O2	0.0556 (4)	0.2787 (2)	0.74212 (14)	0.0256 (7)	
O3	−0.1953 (3)	0.1336 (2)	0.47839 (12)	0.0134 (5)	
O4	−0.2014 (3)	−0.0418 (2)	0.44161 (14)	0.0182 (6)	
O5	−0.5601 (4)	−0.0408 (2)	0.76338 (15)	0.0286 (7)	
O6	0.3897 (3)	0.2655 (2)	0.40749 (14)	0.0174 (6)	
O7	−0.0443 (3)	0.3152 (2)	0.58962 (15)	0.0198 (6)	
H71	−0.026 (5)	0.325 (4)	0.6372 (12)	0.045 (15)*	
H72	0.018 (4)	0.355 (3)	0.571 (2)	0.030 (13)*	
O8	−0.1305 (4)	−0.0824 (2)	0.59515 (15)	0.0217 (6)	
H81	−0.153 (4)	−0.078 (3)	0.5467 (11)	0.012 (9)*	
H82	−0.204 (4)	−0.125 (3)	0.601 (3)	0.042 (15)*	
N1	−0.2899 (3)	0.1512 (2)	0.62260 (15)	0.0126 (6)	
N2	−0.4635 (3)	0.0913 (2)	0.84909 (15)	0.0157 (7)	
N3	0.1205 (3)	0.0828 (2)	0.54918 (15)	0.0118 (6)	
N4	0.2885 (3)	0.1367 (2)	0.31866 (15)	0.0139 (6)	
C1	0.0513 (4)	0.1813 (3)	0.75019 (18)	0.0151 (8)	
C2	0.0678 (4)	0.1339 (3)	0.83163 (18)	0.0147 (7)	
C3	0.0873 (4)	0.2070 (4)	0.89380 (19)	0.0196 (8)	
H3	0.0935	0.2818	0.8854	0.023*	
C4	0.0972 (5)	0.1667 (3)	0.9687 (2)	0.0232 (9)	
H4	0.1122	0.2141	1.0107	0.028*	
C5	0.0848 (5)	0.0566 (3)	0.97913 (19)	0.0191 (8)	
C6	0.0646 (4)	−0.0172 (3)	0.91911 (19)	0.0199 (8)	
H6	0.0569	−0.0918	0.9281	0.024*	
C7	0.0559 (4)	0.0232 (3)	0.84411 (19)	0.0177 (8)	
H7	0.0421	−0.0250	0.8025	0.021*	
C8	−0.2081 (4)	0.0570 (3)	0.42829 (19)	0.0142 (8)	
C9	−0.2320 (4)	0.0953 (3)	0.34487 (18)	0.0128 (7)	
C10	−0.2276 (4)	0.2049 (3)	0.32745 (18)	0.0149 (7)	
H10	−0.2114	0.2559	0.3673	0.018*	
C11	−0.2466 (4)	0.2406 (3)	0.2520 (2)	0.0160 (8)	
H11	−0.2445	0.3148	0.2405	0.019*	
C12	−0.2690 (5)	0.1626 (3)	0.19390 (19)	0.0183 (8)	
C13	−0.2709 (5)	0.0525 (3)	0.2096 (2)	0.0204 (9)	
H13	−0.2847	0.0014	0.1697	0.024*	
C14	−0.2517 (4)	0.0190 (3)	0.28592 (19)	0.0179 (8)	
H14	−0.2522	−0.0552	0.2975	0.021*	
C15	−0.3960 (4)	0.2245 (3)	0.58675 (17)	0.0124 (7)	
H15	−0.3736	0.2599	0.5434	0.015*	
C16	−0.5378 (5)	0.2501 (3)	0.61129 (19)	0.0151 (8)	
H16	−0.6095	0.3020	0.5850	0.018*	
C17	−0.5719 (4)	0.1976 (3)	0.67556 (18)	0.0153 (7)	
H17	−0.6658	0.2146	0.6938	0.018*	
C18	−0.4640 (4)	0.1192 (3)	0.71239 (17)	0.0131 (7)	
C19	−0.3252 (4)	0.0974 (3)	0.68331 (17)	0.0130 (7)	
H19	−0.2539	0.0434	0.7068	0.016*	
C20	−0.4993 (4)	0.0495 (3)	0.7767 (2)	0.0160 (8)	
C21	−0.5022 (5)	0.0272 (3)	0.9134 (2)	0.0197 (9)	
H21A	−0.5973	−0.0177	0.8944	0.024*	
H21B	−0.5295	0.0769	0.9518	0.024*	
C22	−0.3628 (5)	−0.0458 (4)	0.9514 (2)	0.0278 (10)	
H22A	−0.3900	−0.0801	0.9961	0.042*	
H22B	−0.2659	−0.0025	0.9667	0.042*	
H22C	−0.3440	−0.1009	0.9154	0.042*	
C23	−0.3767 (5)	0.1954 (3)	0.86888 (19)	0.0196 (8)	
H23A	−0.3204	0.2151	0.8278	0.024*	
H23B	−0.2952	0.1857	0.9159	0.024*	
C24	−0.4906 (6)	0.2879 (4)	0.8806 (2)	0.0303 (10)	
H24A	−0.4283	0.3534	0.8946	0.045*	
H24B	−0.5470	0.2687	0.9210	0.045*	
H24C	−0.5684	0.3001	0.8335	0.045*	
C25	0.2162 (4)	0.0000 (3)	0.58143 (17)	0.0128 (7)	
H25	0.1921	−0.0352	0.6245	0.015*	
C26	0.3494 (4)	−0.0343 (3)	0.55230 (18)	0.0152 (8)	
H26	0.4136	−0.0921	0.5753	0.018*	
C27	0.3865 (4)	0.0181 (3)	0.48863 (18)	0.0134 (7)	
H27	0.4752	−0.0041	0.4679	0.016*	
C28	0.2882 (4)	0.1048 (3)	0.45606 (18)	0.0124 (7)	
C29	0.1590 (4)	0.1348 (3)	0.48858 (17)	0.0124 (7)	
H29	0.0952	0.1939	0.4676	0.015*	
C30	0.3275 (4)	0.1746 (3)	0.3915 (2)	0.0129 (8)	
C31	0.1984 (5)	0.0353 (3)	0.2969 (2)	0.0186 (8)	
H31A	0.1083	0.0501	0.2542	0.022*	
H31B	0.1530	0.0098	0.3402	0.022*	
C32	0.3040 (5)	−0.0541 (3)	0.2733 (2)	0.0265 (9)	
H32A	0.2398	−0.1190	0.2602	0.040*	
H32B	0.3930	−0.0695	0.3154	0.040*	
H32C	0.3462	−0.0304	0.2293	0.040*	
C33	0.3236 (4)	0.2055 (4)	0.25605 (19)	0.0191 (8)	
H33A	0.3514	0.1591	0.2160	0.023*	
H33B	0.4174	0.2512	0.2759	0.023*	
C34	0.1815 (5)	0.2775 (4)	0.2212 (2)	0.0269 (9)	
H34A	0.2118	0.3232	0.1821	0.040*	
H34B	0.1518	0.3224	0.2608	0.040*	
H34C	0.0903	0.2325	0.1984	0.040*	

### Atomic displacement parameters (Å^2^)

**Table:**

	*U*^11^	*U*^22^	*U*^33^	*U*^12^	*U*^13^	*U*^23^
Br1	0.0422 (3)	0.0408 (3)	0.01315 (17)	0.0001 (2)	0.00294 (16)	0.00690 (18)
Br2	0.0464 (3)	0.0329 (2)	0.01177 (17)	−0.0012 (2)	0.00189 (16)	0.00394 (17)
Cu1	0.01055 (19)	0.0157 (2)	0.00941 (18)	0.0017 (2)	0.00319 (15)	0.00015 (17)
O1	0.0124 (11)	0.0200 (15)	0.0102 (11)	0.0010 (11)	0.0022 (9)	−0.0007 (9)
O2	0.0405 (18)	0.0236 (16)	0.0123 (13)	−0.0064 (14)	0.0046 (12)	0.0003 (11)
O3	0.0130 (12)	0.0162 (13)	0.0112 (11)	0.0004 (11)	0.0026 (9)	−0.0036 (10)
O4	0.0232 (15)	0.0117 (13)	0.0201 (13)	−0.0012 (11)	0.0054 (11)	0.0032 (10)
O5	0.0404 (19)	0.0231 (16)	0.0244 (15)	−0.0148 (14)	0.0112 (13)	−0.0034 (12)
O6	0.0201 (15)	0.0150 (14)	0.0178 (13)	−0.0043 (12)	0.0053 (11)	−0.0018 (10)
O7	0.0217 (15)	0.0183 (15)	0.0198 (14)	−0.0056 (13)	0.0051 (12)	0.0006 (12)
O8	0.0232 (15)	0.0228 (15)	0.0182 (14)	−0.0045 (14)	0.0018 (12)	0.0013 (12)
N1	0.0114 (14)	0.0144 (16)	0.0122 (13)	−0.0002 (12)	0.0028 (11)	0.0004 (11)
N2	0.0158 (15)	0.0213 (18)	0.0106 (14)	−0.0042 (14)	0.0036 (12)	0.0008 (12)
N3	0.0136 (14)	0.0119 (14)	0.0105 (13)	−0.0009 (13)	0.0037 (11)	−0.0001 (11)
N4	0.0119 (14)	0.0156 (16)	0.0141 (14)	−0.0043 (14)	0.0024 (11)	0.0006 (12)
C1	0.0138 (19)	0.024 (2)	0.0070 (15)	0.0050 (16)	0.0019 (13)	−0.0017 (14)
C2	0.0122 (16)	0.021 (2)	0.0112 (15)	0.0020 (17)	0.0016 (13)	0.0008 (15)
C3	0.019 (2)	0.020 (2)	0.0192 (17)	−0.0008 (19)	0.0028 (15)	0.0008 (17)
C4	0.030 (2)	0.028 (2)	0.0111 (17)	0.0016 (19)	0.0016 (16)	−0.0051 (15)
C5	0.0164 (19)	0.031 (2)	0.0080 (16)	0.0027 (18)	−0.0032 (14)	0.0059 (15)
C6	0.0165 (19)	0.024 (2)	0.0194 (18)	−0.0006 (18)	0.0040 (15)	0.0042 (16)
C7	0.0184 (19)	0.022 (2)	0.0132 (16)	−0.0016 (17)	0.0035 (14)	−0.0014 (14)
C8	0.0073 (17)	0.020 (2)	0.0163 (17)	0.0011 (16)	0.0038 (14)	0.0015 (15)
C9	0.0141 (17)	0.014 (2)	0.0112 (16)	0.0033 (15)	0.0043 (13)	−0.0003 (13)
C10	0.0196 (18)	0.0157 (18)	0.0101 (15)	0.0033 (17)	0.0046 (13)	−0.0020 (15)
C11	0.0136 (18)	0.0130 (19)	0.0213 (19)	0.0040 (15)	0.0028 (15)	0.0017 (14)
C12	0.020 (2)	0.027 (2)	0.0069 (15)	−0.0013 (17)	0.0008 (14)	0.0031 (14)
C13	0.027 (2)	0.018 (2)	0.0164 (18)	−0.0051 (18)	0.0032 (16)	−0.0047 (15)
C14	0.021 (2)	0.016 (2)	0.0153 (16)	−0.0035 (16)	0.0000 (15)	−0.0016 (14)
C15	0.0152 (18)	0.0125 (19)	0.0087 (15)	−0.0014 (15)	0.0007 (13)	−0.0011 (13)
C16	0.0189 (19)	0.0131 (18)	0.0127 (16)	−0.0014 (16)	0.0014 (14)	−0.0032 (13)
C17	0.0127 (17)	0.0168 (19)	0.0168 (16)	−0.0004 (16)	0.0040 (14)	−0.0091 (15)
C18	0.0157 (16)	0.0136 (17)	0.0098 (15)	−0.0061 (18)	0.0022 (12)	−0.0027 (15)
C19	0.0160 (17)	0.0132 (19)	0.0096 (15)	−0.0006 (14)	0.0021 (13)	0.0012 (12)
C20	0.0085 (17)	0.0154 (19)	0.026 (2)	0.0001 (16)	0.0071 (15)	0.0049 (16)
C21	0.022 (2)	0.025 (2)	0.0144 (17)	−0.0032 (17)	0.0085 (15)	0.0047 (15)
C22	0.028 (2)	0.031 (2)	0.026 (2)	0.0022 (19)	0.0084 (18)	0.0126 (17)
C23	0.021 (2)	0.025 (2)	0.0132 (16)	−0.0096 (18)	0.0050 (15)	−0.0036 (16)
C24	0.042 (3)	0.027 (2)	0.022 (2)	−0.007 (2)	0.0066 (19)	−0.0067 (17)
C25	0.0144 (18)	0.0114 (17)	0.0125 (15)	−0.0026 (16)	0.0028 (13)	−0.0029 (14)
C26	0.0187 (19)	0.0116 (18)	0.0137 (16)	0.0009 (15)	−0.0010 (14)	−0.0005 (13)
C27	0.0172 (18)	0.0125 (19)	0.0117 (15)	−0.0009 (16)	0.0055 (13)	−0.0025 (13)
C28	0.0163 (17)	0.0079 (19)	0.0134 (16)	−0.0025 (15)	0.0039 (13)	−0.0035 (13)
C29	0.0144 (16)	0.0095 (17)	0.0125 (15)	0.0004 (16)	0.0009 (13)	−0.0004 (14)
C30	0.0129 (18)	0.0138 (18)	0.0134 (16)	0.0028 (15)	0.0057 (14)	−0.0012 (14)
C31	0.0188 (19)	0.024 (2)	0.0116 (16)	−0.0060 (17)	−0.0005 (14)	0.0002 (14)
C32	0.037 (3)	0.020 (2)	0.0215 (19)	0.0020 (19)	0.0039 (18)	0.0010 (16)
C33	0.0181 (19)	0.024 (2)	0.0168 (16)	−0.0004 (18)	0.0085 (15)	0.0043 (17)
C34	0.023 (2)	0.035 (2)	0.022 (2)	0.008 (2)	0.0038 (17)	0.0130 (17)

### Geometric parameters (Å, °)

**Table:**

Br1—C5	1.904 (3)	C15—C16	1.377 (5)
Br2—C12	1.901 (3)	C15—H15	0.9300
Cu1—O1	1.985 (2)	C16—H16	0.9300
Cu1—O3	1.979 (2)	C17—C16	1.382 (5)
Cu1—O7	2.377 (3)	C17—C18	1.386 (5)
Cu1—O8	2.543 (3)	C17—H17	0.9300
Cu1—N1	1.996 (3)	C18—C19	1.385 (4)
Cu1—N3	1.998 (3)	C18—C20	1.495 (5)
O1—C1	1.289 (4)	C19—H19	0.9300
O2—C1	1.200 (5)	C20—N2	1.354 (4)
O3—C8	1.277 (4)	C21—N2	1.467 (4)
O4—C8	1.229 (4)	C21—C22	1.515 (5)
O5—C20	1.217 (5)	C21—H21A	0.9700
O6—C30	1.235 (4)	C21—H21B	0.9700
O7—H71	0.831 (19)	C22—H22A	0.9600
O7—H72	0.823 (19)	C22—H22B	0.9600
O8—H81	0.841 (18)	C22—H22C	0.9600
O8—H82	0.826 (19)	C23—N2	1.472 (5)
N1—C19	1.339 (4)	C23—C24	1.518 (6)
N3—C29	1.337 (4)	C23—H23A	0.9700
N4—C30	1.343 (4)	C23—H23B	0.9700
N4—C31	1.462 (5)	C24—H24A	0.9600
N4—C33	1.464 (4)	C24—H24B	0.9600
C2—C1	1.531 (4)	C24—H24C	0.9600
C2—C7	1.377 (5)	C25—N3	1.344 (5)
C3—C2	1.399 (5)	C25—C26	1.382 (5)
C3—C4	1.397 (5)	C25—H25	0.9300
C3—H3	0.9300	C26—H26	0.9300
C4—H4	0.9300	C27—C26	1.381 (5)
C5—C4	1.364 (6)	C27—H27	0.9300
C5—C6	1.376 (5)	C28—C27	1.393 (5)
C6—C7	1.401 (5)	C28—C29	1.371 (4)
C6—H6	0.9300	C29—H29	0.9300
C7—H7	0.9300	C30—C28	1.511 (5)
C8—C9	1.520 (4)	C31—C32	1.514 (5)
C10—C9	1.376 (5)	C31—H31A	0.9700
C10—H10	0.9300	C31—H31B	0.9700
C11—C10	1.381 (5)	C32—H32A	0.9600
C11—C12	1.385 (5)	C32—H32B	0.9600
C11—H11	0.9300	C32—H32C	0.9600
C13—C12	1.374 (6)	C33—C34	1.507 (5)
C13—H13	0.9300	C33—H33A	0.9700
C14—C9	1.382 (4)	C33—H33B	0.9700
C14—C13	1.387 (5)	C34—H34A	0.9600
C14—H14	0.9300	C34—H34B	0.9600
C15—N1	1.329 (4)	C34—H34C	0.9600
			
O1—Cu1—O7	92.38 (10)	C16—C17—H17	120.5
O1—Cu1—N1	89.72 (10)	C18—C17—H17	120.5
O1—Cu1—N3	91.99 (10)	C17—C18—C20	122.8 (3)
O3—Cu1—O1	178.70 (11)	C19—C18—C17	118.3 (3)
O3—Cu1—O7	88.92 (10)	C19—C18—C20	118.6 (3)
O3—Cu1—N1	90.39 (10)	N1—C19—C18	122.4 (3)
O3—Cu1—N3	87.81 (10)	N1—C19—H19	118.8
N1—Cu1—O7	86.50 (10)	C18—C19—H19	118.8
N1—Cu1—N3	175.65 (12)	O5—C20—N2	122.0 (3)
N3—Cu1—O7	97.43 (11)	O5—C20—C18	120.6 (3)
C1—O1—Cu1	127.5 (2)	N2—C20—C18	117.5 (3)
C8—O3—Cu1	125.5 (2)	N2—C21—C22	112.8 (3)
Cu1—O7—H71	99 (3)	N2—C21—H21A	109.0
Cu1—O7—H72	132 (3)	N2—C21—H21B	109.0
H71—O7—H72	108 (5)	C22—C21—H21A	109.0
H81—O8—H82	99 (4)	C22—C21—H21B	109.0
C15—N1—Cu1	120.9 (2)	H21A—C21—H21B	107.8
C15—N1—C19	118.6 (3)	C21—C22—H22A	109.5
C19—N1—Cu1	120.4 (2)	C21—C22—H22B	109.5
C20—N2—C21	119.2 (3)	C21—C22—H22C	109.5
C20—N2—C23	123.8 (3)	H22A—C22—H22B	109.5
C21—N2—C23	116.9 (3)	H22A—C22—H22C	109.5
C25—N3—Cu1	120.6 (2)	H22B—C22—H22C	109.5
C29—N3—Cu1	120.7 (2)	N2—C23—C24	112.5 (3)
C29—N3—C25	118.6 (3)	N2—C23—H23A	109.1
C30—N4—C31	123.9 (3)	N2—C23—H23B	109.1
C30—N4—C33	118.5 (3)	C24—C23—H23A	109.1
C31—N4—C33	117.3 (3)	C24—C23—H23B	109.1
O1—C1—C2	113.1 (3)	H23A—C23—H23B	107.8
O2—C1—O1	127.7 (3)	C23—C24—H24A	109.5
O2—C1—C2	119.2 (3)	C23—C24—H24B	109.5
C3—C2—C1	118.0 (3)	C23—C24—H24C	109.5
C7—C2—C1	121.8 (3)	H24A—C24—H24B	109.5
C7—C2—C3	120.2 (3)	H24A—C24—H24C	109.5
C2—C3—H3	120.2	H24B—C24—H24C	109.5
C4—C3—C2	119.5 (4)	N3—C25—C26	121.8 (3)
C4—C3—H3	120.2	N3—C25—H25	119.1
C3—C4—H4	120.5	C26—C25—H25	119.1
C5—C4—C3	118.9 (4)	C25—C26—H26	120.3
C5—C4—H4	120.5	C27—C26—C25	119.4 (3)
C4—C5—Br1	119.3 (3)	C27—C26—H26	120.3
C4—C5—C6	122.9 (3)	C26—C27—C28	118.6 (3)
C6—C5—Br1	117.8 (3)	C26—C27—H27	120.7
C5—C6—C7	118.1 (4)	C28—C27—H27	120.7
C5—C6—H6	120.9	C27—C28—C30	122.8 (3)
C7—C6—H6	120.9	C29—C28—C27	118.7 (3)
C2—C7—C6	120.4 (3)	C29—C28—C30	118.2 (3)
C2—C7—H7	119.8	N3—C29—C28	122.9 (3)
C6—C7—H7	119.8	N3—C29—H29	118.6
O3—C8—C9	114.9 (3)	C28—C29—H29	118.6
O4—C8—O3	126.4 (3)	O6—C30—N4	122.7 (3)
O4—C8—C9	118.7 (3)	O6—C30—C28	118.7 (3)
C10—C9—C8	120.8 (3)	N4—C30—C28	118.7 (3)
C10—C9—C14	119.5 (3)	N4—C31—C32	112.7 (3)
C14—C9—C8	119.6 (3)	N4—C31—H31A	109.1
C9—C10—C11	121.3 (3)	N4—C31—H31B	109.1
C9—C10—H10	119.3	C32—C31—H31A	109.1
C11—C10—H10	119.3	C32—C31—H31B	109.1
C10—C11—C12	118.0 (3)	H31A—C31—H31B	107.8
C10—C11—H11	121.0	C31—C32—H32A	109.5
C12—C11—H11	121.0	C31—C32—H32B	109.5
C11—C12—Br2	118.6 (3)	C31—C32—H32C	109.5
C13—C12—Br2	119.3 (3)	H32A—C32—H32B	109.5
C13—C12—C11	122.0 (3)	H32A—C32—H32C	109.5
C12—C13—C14	118.7 (3)	H32B—C32—H32C	109.5
C12—C13—H13	120.6	N4—C33—C34	112.5 (3)
C14—C13—H13	120.6	N4—C33—H33A	109.1
C9—C14—C13	120.4 (3)	N4—C33—H33B	109.1
C9—C14—H14	119.8	C34—C33—H33A	109.1
C13—C14—H14	119.8	C34—C33—H33B	109.1
N1—C15—C16	122.6 (3)	H33A—C33—H33B	107.8
N1—C15—H15	118.7	C33—C34—H34A	109.5
C16—C15—H15	118.7	C33—C34—H34B	109.5
C15—C16—C17	118.9 (3)	C33—C34—H34C	109.5
C15—C16—H16	120.5	H34A—C34—H34B	109.5
C17—C16—H16	120.5	H34A—C34—H34C	109.5
C16—C17—C18	119.0 (3)	H34B—C34—H34C	109.5
			
O7—Cu1—O1—C1	23.4 (3)	C5—C6—C7—C2	0.1 (5)
N1—Cu1—O1—C1	−63.1 (3)	O3—C8—C9—C10	4.5 (5)
N3—Cu1—O1—C1	120.9 (3)	O3—C8—C9—C14	−178.2 (3)
O7—Cu1—O3—C8	155.5 (3)	O4—C8—C9—C10	−174.9 (4)
N1—Cu1—O3—C8	−118.0 (3)	O4—C8—C9—C14	2.4 (5)
N3—Cu1—O3—C8	58.1 (3)	C11—C10—C9—C8	179.0 (3)
O1—Cu1—N1—C15	141.7 (3)	C11—C10—C9—C14	1.7 (6)
O1—Cu1—N1—C19	−37.7 (2)	C12—C11—C10—C9	−0.6 (5)
O3—Cu1—N1—C15	−39.6 (3)	C10—C11—C12—Br2	179.1 (3)
O3—Cu1—N1—C19	141.0 (3)	C10—C11—C12—C13	−0.7 (6)
O7—Cu1—N1—C15	49.3 (2)	C14—C13—C12—Br2	−178.9 (3)
O7—Cu1—N1—C19	−130.1 (3)	C14—C13—C12—C11	0.8 (6)
O1—Cu1—N3—C25	43.0 (3)	C13—C14—C9—C8	−178.9 (3)
O1—Cu1—N3—C29	−140.1 (2)	C13—C14—C9—C10	−1.5 (5)
O3—Cu1—N3—C25	−135.7 (3)	C9—C14—C13—C12	0.3 (6)
O3—Cu1—N3—C29	41.2 (3)	C16—C15—N1—C19	2.4 (5)
O7—Cu1—N3—C25	135.7 (2)	C16—C15—N1—Cu1	−177.0 (3)
O7—Cu1—N3—C29	−47.5 (3)	N1—C15—C16—C17	−0.2 (5)
Cu1—O1—C1—O2	−27.2 (6)	C18—C17—C16—C15	−1.3 (5)
Cu1—O1—C1—C2	152.3 (2)	C16—C17—C18—C19	0.5 (5)
Cu1—O3—C8—O4	25.0 (5)	C16—C17—C18—C20	−173.0 (3)
Cu1—O3—C8—C9	−154.4 (2)	C17—C18—C19—N1	1.9 (5)
Cu1—N1—C19—C18	176.1 (2)	C20—C18—C19—N1	175.6 (3)
C15—N1—C19—C18	−3.3 (5)	C17—C18—C20—O5	94.2 (5)
Cu1—N3—C29—C28	−174.5 (3)	C17—C18—C20—N2	−84.9 (4)
C25—N3—C29—C28	2.5 (5)	C19—C18—C20—O5	−79.3 (4)
C31—N4—C30—O6	−172.8 (3)	C19—C18—C20—N2	101.7 (4)
C31—N4—C30—C28	4.9 (5)	O5—C20—N2—C21	−1.4 (5)
C33—N4—C30—O6	1.0 (5)	O5—C20—N2—C23	174.3 (3)
C33—N4—C30—C28	178.7 (3)	C18—C20—N2—C21	177.6 (3)
C30—N4—C31—C32	−109.2 (4)	C18—C20—N2—C23	−6.7 (5)
C33—N4—C31—C32	76.9 (4)	C22—C21—N2—C20	90.9 (4)
C30—N4—C33—C34	−92.0 (4)	C22—C21—N2—C23	−85.0 (4)
C31—N4—C33—C34	82.2 (4)	C24—C23—N2—C20	102.7 (4)
C3—C2—C1—O1	−179.5 (3)	C24—C23—N2—C21	−81.5 (4)
C3—C2—C1—O2	0.0 (5)	C26—C25—N3—Cu1	175.1 (2)
C7—C2—C1—O1	−2.7 (5)	C26—C25—N3—C29	−1.8 (5)
C7—C2—C1—O2	176.8 (4)	N3—C25—C26—C27	0.4 (5)
C1—C2—C7—C6	−177.2 (3)	C28—C27—C26—C25	0.5 (5)
C3—C2—C7—C6	−0.5 (5)	C29—C28—C27—C26	0.1 (5)
C4—C3—C2—C1	177.9 (3)	C30—C28—C27—C26	173.8 (3)
C4—C3—C2—C7	1.0 (5)	C27—C28—C29—N3	−1.6 (5)
C2—C3—C4—C5	−1.2 (6)	C30—C28—C29—N3	−175.6 (3)
Br1—C5—C4—C3	−179.5 (3)	O6—C30—C28—C27	−101.2 (4)
C6—C5—C4—C3	0.8 (7)	O6—C30—C28—C29	72.5 (4)
Br1—C5—C6—C7	−180.0 (3)	N4—C30—C28—C27	81.0 (4)
C4—C5—C6—C7	−0.3 (6)	N4—C30—C28—C29	−105.3 (4)

### Hydrogen-bond geometry (Å, °)

**Table:**

*D*—H···*A*	*D*—H	H···*A*	*D*···*A*	*D*—H···*A*
O7—H71···O2	0.83 (2)	1.93 (3)	2.692 (4)	154 (5)
O7—H72···O4^i^	0.83 (4)	2.04 (4)	2.834 (4)	163 (3)
O8—H81···O4	0.84 (2)	1.88 (2)	2.702 (4)	170 (4)
O8—H82···O6^ii^	0.82 (4)	2.05 (4)	2.848 (4)	168 (5)
C11—H11···O5^iii^	0.93	2.41	3.106 (5)	133
C16—H16···O4^iii^	0.93	2.46	3.359 (5)	162
C26—H26···O6^iv^	0.93	2.37	3.261 (4)	160

Symmetry codes: (i) −*x*, *y*+1/2, −*z*+1; (ii) −*x*, *y*−1/2, −*z*+1; (iii) −*x*−1, *y*+1/2, −*z*+1; (iv) −*x*+1, *y*−1/2, −*z*+1.

## References

[bb1] Allen, F. H., Kennard, O., Watson, D. G., Brammer, L., Orpen, A. G. & Taylor, R. (1987). *J. Chem. Soc. Perkin Trans. 2*, pp. S1–19.

[bb2] Bigoli, F., Braibanti, A., Pellinghelli, M. A. & Tiripicchio, A. (1972). *Acta Cryst.* B**28**, 962–966.

[bb3] Bruker (2005). *SADABS* Bruker AXS Inc., Madison, Wisconsin, USA.

[bb4] Bruker (2007). *APEX2* and *SAINT* Bruker AXS Inc., Madison, Wisconsin, USA.

[bb5] Farrugia, L. J. (1997). *J. Appl. Cryst.* **30**, 565.

[bb6] Farrugia, L. J. (1999). *J. Appl. Cryst.* **32**, 837–838.

[bb7] Flack, H. D. (1983). *Acta Cryst.* A**39**, 876–881.

[bb8] Hökelek, T., Dal, H., Tercan, B., Özbek, F. E. & Necefoğlu, H. (2009*a*). *Acta Cryst.* E**65**, m466–m467. 10.1107/S1600536809011209 PMC2968958 21582397

[bb9] Hökelek, T., Dal, H., Tercan, B., Özbek, F. E. & Necefoğlu, H. (2009*b*). *Acta Cryst.* E**65**, m607–m608. 10.1107/S1600536809015645 PMC2977639 21583825

[bb10] Hökelek, T., Gündüz, H. & Necefoğlu, H. (1996). *Acta Cryst.* C**52**, 2470–2473.

[bb11] Hökelek, T. & Necefoğlu, H. (1998). *Acta Cryst.* C**54**, 1242–1244.

[bb12] Hökelek, T. & Necefoğlu, H. (2007). *Acta Cryst.* E**63**, m821–m823.

[bb13] Krishnamachari, K. A. V. R. (1974). *Am. J. Clin. Nutr.* **27**, 108–111. 10.1093/ajcn/27.2.108 4812927

[bb14] Necefoğlu, H., Maracı, A., Özbek, F. E., Tercan, B. & Hökelek, T. (2011). *Acta Cryst.* E**67**, m619–m620. 10.1107/S1600536811014188 PMC3089307 21754332

[bb15] Sheldrick, G. M. (2008). *Acta Cryst.* A**64**, 112–122. 10.1107/S0108767307043930 18156677

[bb16] Spek, A. L. (2009). *Acta Cryst.* D**65**, 148–155. 10.1107/S090744490804362X PMC2631630 19171970

